# Supplementary material for: PARE: A framework for removal of confounding effects from any distance-based dimension reduction method
Source: PLoS Comput Biol. 2024 Jul 10;20(7):e1012241. doi: 10.1371/journal.pcbi.1012241 (PMC11262650; doi:10.1371/journal.pcbi.1012241)
Supplement: S1 Text — (PDF) [file pcbi.1012241.s001.pdf]

Supplementary Materials for PARE: A framework for removal of confounding effects from any distance-based dimension reduction method

Andrew A. Chen[1,*], Kelly Clark[2], Blake Dewey[4], Anna DuVal[4], Nicole Pellegrini[4], Govind Nair[5], Youmna Jalkh[6], Samar Khalil[6], Jon Zurawski[6], Peter Calabresi[4], Daniel Reich[5], Rohit Bakshi[6,7], Haochang Shou[2,3,¶], Russell T. Shinohara[2,3,¶], Alzheimer's Disease Neuroimaging Initiative , and North American Imaging in Multiple Sclerosis Cooperative

[1] *Department of Public Health Sciences, Medical University of South Carolina, Charleston, South Carolina, United States of America*

[2] *Penn Statistics in Imaging and Visualization Center, Department of Biostatistics, Epidemiology, and Informatics, University of Pennsylvania, Philadelphia, Pennsylvania, United States of America*

[3] *Center for Biomedical Image Computing and Analytics, University of Pennsylvania, Philadelphia, Pennsylvania, United States of America*

[4] *Department of Neurology, Johns Hopkins University School of Medicine, Baltimore, Maryland, United States of America*

[5] *Translational Neuroradiology Section, National Institute of Neurological Disorders and Stroke, National Institutes of Health, Bethesda, Maryland, United States of America*

[6] *Department of Neurology, Brigham and Women's Hospital, Harvard Medical School, Boston, Masschusetts, United States of America*

[7] *Department of Radiology, Brigham and Women's Hospital, Harvard Medical School, Boston, Massachusetts*

[¶] *Equal contribution*

June 14, 2024

***Correspondence: Andrew A. Chen, chenandr@musc.edu**

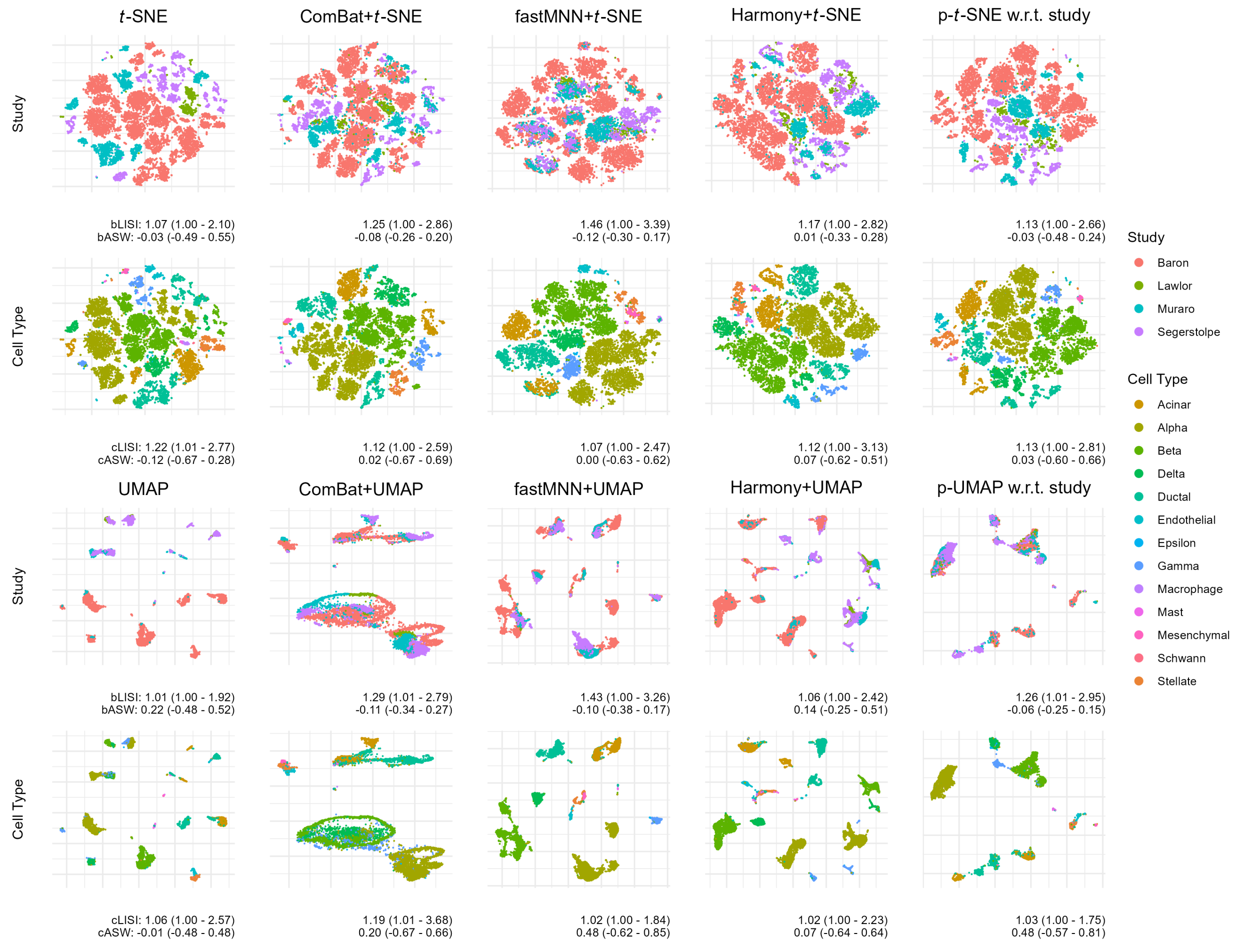

Fig A: **Partial embeddings versus harmonization followed by dimension reduction for batch correction.** Harmonization is performed using ComBat, fastMNN, or Harmony and compared to partial embeddings. Our comparison is performed in single-cell RNA-sequencing measurements from 13,369 human pancreatic cells across four studies. The original counts data is log-normalized and reduced to 2,000 highly variable genes. Local inverse Simpson's index and average silhouette width are computed for each cell for batch (bLISI, bASW) and cell type (cLISI, cASW) with the median, 2.5% quantile, and 97.5% quantile shown. Higher bLISI and lower bASW indicate greater integration across batches. Lower cLISI and higher cASW indicate greater separation between cell types. Partial *t*-SNE (p-*t*-SNE) and partial UMAP (p-UMAP) adjust for batch effects. All *t*-SNE embeddings have a perplexity of 10 and UMAP embeddings use 15 nearest neighbors.

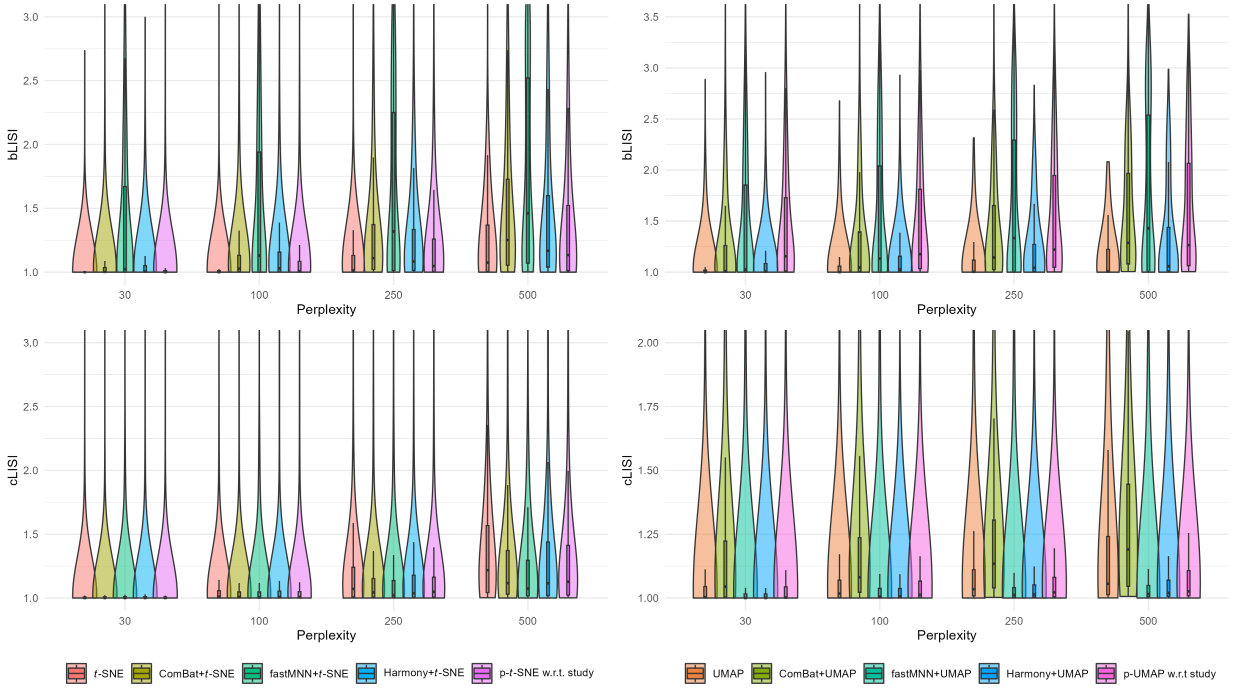

Fig B: **Quantitative comparisons for partial embeddings versus harmonization using local inverse Simpson's index across multiple perplexity values.** Local inverse Simpson's index for batch (bLISI) and cell type (cLISI) are computed on log-normalized single-cell RNA-sequencing data from 13,369 human pancreatic cells across four studies. bLISI and cLISI are computed across multiple perplexity values, which capture different neighborhood sizes around each cell. Higher bLISI corresponds to better batch adjustment performance and lower cLISI indicates greater separation between cell types.

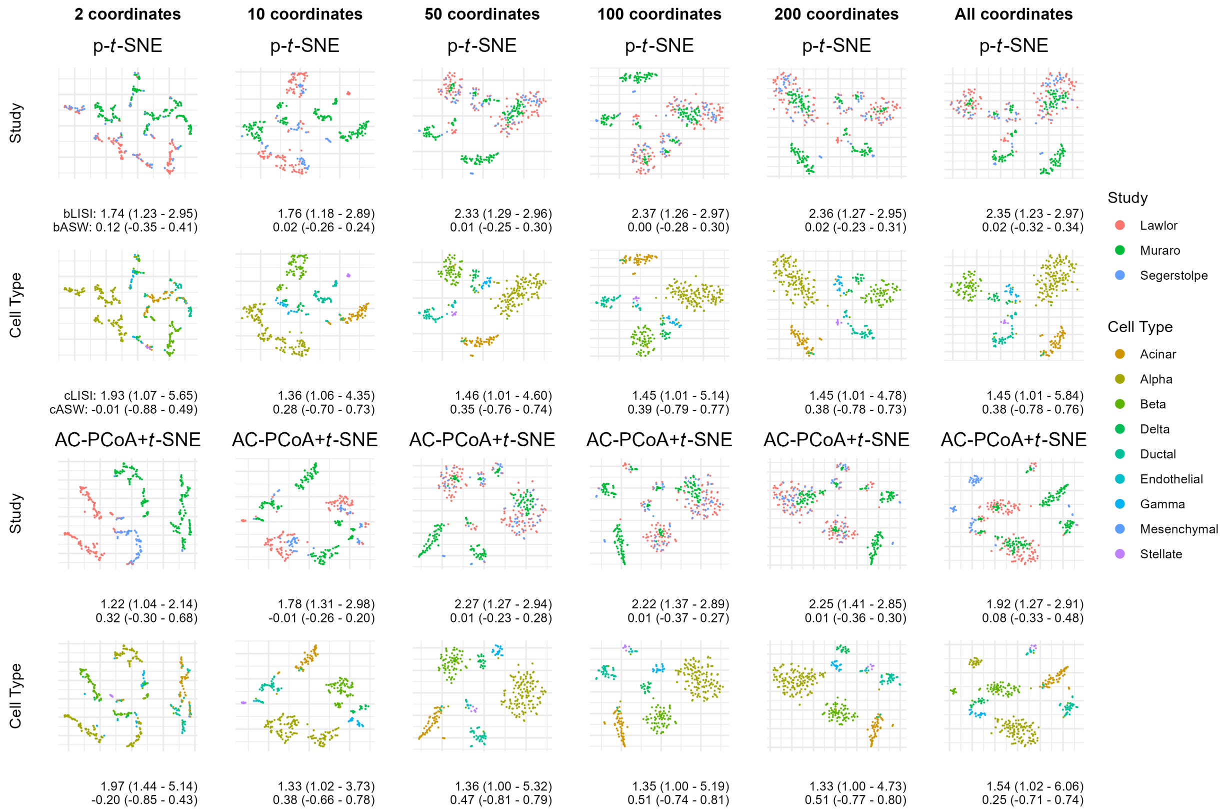

Fig C: **Partial embeddings versus Adjustment for Confounding factors using Principal Coordinate Analysis (AC-PCoA) followed by t-SNE for batch correction.** We compare these methods across varying number of principal coordinates. Our comparison is performed in single-cell RNA-sequencing measurements from 13,369 human pancreatic cells across four studies. The original counts data is log-normalized and reduced to 2,000 highly variable genes. Local inverse Simpson's index and average silhouette width are computed for each cell for batch (bLISI, bASW) and cell type (cLISI, cASW) with the median, 2.5% quantile, and 97.5% quantile shown. Higher bLISI and lower bASW indicate greater integration across batches. Lower cLISI and higher cASW indicate greater separation between cell types. Partial t-SNE (p-t-SNE) and partial UMAP (p-UMAP) adjust for batch effects. All t-SNE embeddings have a perplexity of 10 and UMAP embeddings use 15 nearest neighbors.
