# Supplementary material for: PARE: A framework for removal of confounding effects from any distance-based dimension reduction method
Source: PLoS Comput Biol. 2024 Jul 10;20(7):e1012241. doi: 10.1371/journal.pcbi.1012241 (PMC11262650; doi:10.1371/journal.pcbi.1012241)
Supplement: Attachment — Submitted filename: 231210_scRNA_batch_visualisation.docx [file pcbi.1012241.s002.docx]

The manuscript discusses a methodology, PARE, designed to remove confounding effects for dimensionality reductions techniques that are distance-based, including UMAP and t-SNE. The main contribution of this technique is that it is a general framework for removing confounding effects, for *any* distanced-based dimensionality reduction technique. The generalisability of the approach is in principle attractive.

I have two major conceptual concerns:

1) The first is regarding misrepresentation and biological misinterpretation of lower dimensional visualisation, particularly with t-SNE and UMAP in scRNA-seq [https://doi.org/10.1371/journal.pcbi.1011288] that have been discussed a lot recently in the scRNA-seq community. Some of the leading experts in the field are advocating moving away from such “all-in-one” visualisation techniques in the first place. I think this aspect will need to be talked about explicitly in the manuscript and cited. The above reference has extensive information also regarding data integration and batch correction with respect to visualisation. If PARE suffers from similar issues, which would be my expectation given the above reference (in particular Section 3), this will need to be declared explicitly. Without this issue being resolved, the practical relevance of the work remains unclear.
2) The second concern is about whether or not there is benefit in this approach vs other batch correction techniques that are used not only for visualisation, but also for further downstream analyses, such as differential expression (DE) analysis. Since such approaches correct for batch effects for all types of downstream analyses (including dimensional visualisation), it will need to be demonstrated that PARE performs better when it comes to visualisation alone. Probably a good place to start with such a comparison is to take the top 2-3 best performing batch correction methods from this review [https://doi.org/10.1186/s13059-019-1850-9], as well as more recent techniques, and compare/contrast with the final results of PARE on the case study data sets in this paper. (I have noted that the authors cite/compare PARE to other modified visualisation techniques that aim to correct for batch effect, but the comparison would need to go beyond this as reasoned above.)

Also in the abstract: “Our results show that the PARE framework can remove batch effects in single-cell sequencing.” Evidence is only provided for visualisation, not other downstream applications. Best to add “for reduced dimensional visualisation” or similar, to be specific.

The metric “LISI” used will need to be explained in more depth and justified within the manuscript. Are there other metrics, what are the pros and cons, why was this metric in particular chosen? All comparisons in the work rely on this metric. This information is missing from the manuscript and the reader is merely referred to a citation, which is not sufficient.

The figure captions are merely descriptive of the plots and lack insight and/or conclusions (take home message for the reader). These need to be improved.

There does not seem to be a link to the code on GitHub (or similar). I have not managed to find the code by Googling the name of the authors or title of the manuscript. Please make your software available.
