# Supplementary material for: PARE: A framework for removal of confounding effects from any distance-based dimension reduction method
Source: PLoS Comput Biol. 2024 Jul 10;20(7):e1012241. doi: 10.1371/journal.pcbi.1012241 (PMC11262650; doi:10.1371/journal.pcbi.1012241)
Supplement: Attachment — Submitted filename: Response to Reviewer Comments.docx [file pcbi.1012241.s003.docx]

**Response to Reviewer Comments**

We thank the reviewers for their efforts and insightful comments. Following their suggestions, we have incorporated extensive revisions to the text as well as several new analyses highlighted below:

1. Comparison of PAREs to harmonization via ComBat, fastMNN, and Harmony followed by dimension reduction in Case Study 1 (scRNA-seq data)
2. Application of AC-PCoA and comparison to PAREs across varying numbers of coordinates
3. Addition of the average silhouette width (ASW) as an alternative metric to the local inverse Simpson’s index (LISI) for quantitative evaluations and comparisons
4. Computation and reporting of the LISI and ASW for Case Studies 2 and 3.

Other changes include revisions and sharing of a software implementation in R and several major edits to the primary figures. Our responses are written in blue. We hope that these revisions address the concerns of the reviewers. We appreciate the thought and feedback and believe that the reviewer’s suggestions have greatly improved the manuscript.

**Response to Reviewer #1:**

Reviewer #1: This paper proposes an approach for the removal of confounding sources of variation prior to visualization using dimension reduction methods including t-SNE and UMAP. The method is clearly described and illustrated on three real-world data sets.

Comments:
* The approach for removing the confounding variation assumes a linear relation between the confounders and a Euclidean representation of the data. There is a newer method AC-PCoA (Wang et al. 2022) that allows for non-linear effects of confounders. Is it possible to utilize this framework to construct the covariate-adjusted dissimilarities?

Thank you for this inquiry. After reviewing the AC-PCoA (Wang et al., 2022) method, we have determined that AC-PCoA simultaneously removes confounding effects while estimating the lower-dimensional representation. This estimation procedure yields confounder-adjusted dimensions, without yielding an adjusted dissimilarity. PARE however operates on an adjusted dissimilarity and cannot readily incorporate nonlinear adjustment via AC-PCoA.

However, applying AC-PCoA followed by a *t*-SNE has been explored in the Wang et al., 2022 paper. We perform an additional analysis to compare batch correction using AC-PCoA followed by *t*-SNE versus p-*t*-SNE with respect to study. We describe our new analysis in **Section 3.1**:

“We apply p-t-SNE and p-UMAP to remove batch effect or donor effects in the embeddings. We compare p-t-SNE to other methods that simultaneously remove confounding effects and obtain lower-dimensional representations including projected t-SNE for batch correction (BC-t-SNE, Aliverti et al., 2020) and Adjustment for Confounding factors using Principal Coordinate Analysis (AC-PCoA, Wang et al., 2022), proposed in their original paper. For BC-t-SNE, we set the perplexity at 10 to be similar to our p-t-SNE approach. For AC-PCoA, we compare AC-PCoA followed by t-SNE to p-t-SNE with respect to study across varying numbers of coordinates for both methods. Due to computational complexity of AC-PCoA, we perform these comparisons in a dataset consisting of human pancreatic cell scRNA-seq data from three donors: donor ACCG268 from Lawlor et al. (2017) (136 cells), donor AZ from Segerstolpe et al. (2016) (63 cells), and donor D28 from Muraro et al. (2016) (181 cells)”

Our new results in a subset of the Case Study 1 dataset are highlighted briefly in **Section 4.1** and **Supplementary Figure 3** copied below:

“In a subset of three donors, we additionally compare p-t-SNE to AC-PCoA+t-SNE, showing comparable performance across varying numbers of coordinates (**Supplementary Figure 3**)”

* An alternative approach to removing confounding effects in visualization would be to apply a recommended batch correction method for the given data type (e.g., Harmony or Seurat for single cell data), and then apply t-SNE or UMAP to the resulting data. How does this approach compare to the proposed method?

Thank you for this excellent suggestion. For the specific use case of removing batch effects in Case Study 1, we agree that our method can be compared to existing batch correction methods. However, our method can also be applied for additional use cases, including continuous confounding variables and multiple confounding variables.

In Case Study 1, we pursue additional analyses to compare partial embeddings (PAREs) to applying ComBat, fastMNN, or Harmony prior to dimension reduction. The details of this analysis are included in **Section 3.1**:

“In this setting, our method is also comparable to two-stage approaches where batch correction is performed prior to dimension reduction. These approaches include Combatting atch Effects (ComBat, Johnson et al., 2007), fastMNN (Haghverdi et al., 2018), and Harmony (Korsunsky et al., 2019) which are all widely employed and have been compared recently (Tran et al., 2020; Luecken et al., 2022; Ryu et al., 2023). For our analyses, we compare p-*t*-SNE and p-UMAP to performing ComBat, fastMNN, or Harmony followed by *t*-SNE or UMAP. ComBat is run using the R implementation in the ComBatHarmonization package (https://github.com/Jfortin1/ComBatHarmonization). fastMNN is run with default settings using the batchelor package version 1.19.1 in R. Harmony is performed with default settings on the top 20 principal components using the harmony package version 1.2.0.”

The results of this analysis are summarized in **Section 4.1**, citing new **Supplementary Figures 1 and 2**:

“Compared to batch correction using ComBat, fastMNN, or Harmony followed by dimension reduction, p-*t*-SNE and p-UMAP w.r.t. study demonstrate comparable performance but fastMNN+*t*-SNE and fastMNN+UMAP outperform other methods (**Supplementary Figures 1 and 2**)”

In the **Discussion**, we comment on these findings:

“As an alternative for batch correction, harmonization methods have been extensively used to reduce batch-related confounding in visualization and downstream analyses (Tran et al., 2020; Luecken et al., 2022; Ryu et al., 2023). Compared to harmonization followed by dimension reduction, we find that PAREs can underperform fastMNN followed by *t*-SNE or UMAP. However, PAREs have several use cases beyond batch adjustment since PAREs can handle continuous confounding variables and multiple confounders.”

* The presentation of some of the figures could be improved. In particular, the violin plots in Figure 2 have the same heights across all methods, making it hard to identify potentially interesting differences in performance across methods.

Thank you for this comment, we’ve adjusted the limits of each plot accordingly and the revised version of Figure 2 is copied below:

* Some other figures rely on the reader to carefully squint at many colored dots (e.g., Figure 4). Could a quantitative summary be used here as well to support the qualitative conclusions?

We agree that a quantitative metric here would be appropriate to more clearly delineate the results. In Figure 4, we now include computation and reporting of both the local inverse Simpson’s index (LISI) and average silhouette width (ASW) across observations. This is described in **Sections 3.2 and 3.3**:

“Quantitatively, we evaluate our methods using the ASW and LISI for manufacturer and diagnosis. Higher manufacturer LISI (mLISI) and lower manufacturer ASW (mASW) indicate better batch adjustment performance. Lower diagnosis LISI (dLISI) and higher diagnosis ASW (dASW) indicate better separation between diagnosis groups.” (**Section 3.2**)

“We additionally evaluate our methods using the ASW and LISI for site and subject. Lower site LISI (bLISI) and higher site ASW (bASW) indicate greater separation between sites. Lower subject LISI (sLISI) and higher subject ASW (sASW) indicate greater separation between subjects.” (**Section 3.3**)

The revised **Figure 4** with updated analyses and captions is copied below:

Minor comments/typos:
* p. 2 : “These methods has…” -> “These methods have…”
* p. 4: “Define the …” -> “We define the …”

These typos have been corrected, thank you for pointing them out.

**Response to Reviewer #2:**

Reviewer #2:

The manuscript discusses a methodology, PARE, designed to remove confounding effects for dimensionality reductions techniques that are distance-based, including UMAP and t-SNE. The main contribution of this technique is that it is a general framework for removing confounding effects, for *any* distanced-based dimensionality reduction technique. The generalisability of the approach is in principle attractive.

I have two major conceptual concerns:

1) The first is regarding misrepresentation and biological misinterpretation of lower dimensional visualisation, particularly with t-SNE and UMAP in scRNA-seq [https://doi.org/10.1371/journal.pcbi.1011288] that have been discussed a lot recently in the scRNA-seq community. Some of the leading experts in the field are advocating moving away from such “all-in-one” visualisation techniques in the first place. I think this aspect will need to be talked about explicitly in the manuscript and cited. The above reference has extensive information also regarding data integration and batch correction with respect to visualisation. If PARE suffers from similar issues, which would be my expectation given the above reference (in particular Section 3), this will need to be declared explicitly. Without this issue being resolved, the practical relevance of the work remains unclear.

Thank you for bringing this article to our attention. We agree that embeddings in general are insufficient for many downstream analyses including clustering, validation, and inference. Addressing this issue is beyond the scope of our article, which aims to extend existing embedding approaches. Our method aims to reduce confounding effects in the visualizations, without making any claims of removing batch effects in the original data, which is not adjusted by our method. We believe that our method remains relevant to perform data visualization while accounting for confounding effects, but admit that our method does not address broader issues with embeddings accurately representing the data. For downstream analyses, we highlight results from Wang et al., 2022, showing that adjusted dissimilarity matrices can be used in downstream analyses to reduce confounding while preserving original data properties.

In the **Discussion**, we explicitly note these limitations and discuss alternatives:

“We do not recommend PAREs to be used in place of the original data in downstream analyses or claim that PAREs preserve all data properties, since distance-based embeddings in general have been demonstrated to distort properties of the original data (Chari and Pachter, 2023). For removal of confounding from downstream analyses, the use of an adjusted dissimilarity matrix has been previously explored and demonstrated to reduce confounding in nonparametric analyses while preserving data properties (Wang et al., 2022).”

2) The second concern is about whether or not there is benefit in this approach vs other batch correction techniques that are used not only for visualisation, but also for further downstream analyses, such as differential expression (DE) analysis. Since such approaches correct for batch effects for all types of downstream analyses (including dimensional visualisation), it will need to be demonstrated that PARE performs better when it comes to visualisation alone. Probably a good place to start with such a comparison is to take the top 2-3 best performing batch correction methods from this review [https://doi.org/10.1186/s13059-019-1850-9], as well as more recent techniques, and compare/contrast with the final results of PARE on the case study data sets in this paper. (I have noted that the authors cite/compare PARE to other modified visualisation techniques that aim to correct for batch effect, but the comparison would need to go beyond this as reasoned above.)

Thank you for this suggestion. Our method is distinct from batch correction methods since we simultaneously perform dimension reduction while removing confounding effects. These confounding variables can be continuous, categorical, or a mix of both. For the specific use case of removing batch effects in Case Study 1, we agree that our method can be compared to existing batch correction methods.

As a result, we pursue additional analyses to compare partial embeddings (PAREs) to applying ComBat, fastMNN, or Harmony prior to dimension reduction. The details of this analysis are included in **Section 3.1**:

“In this setting, our method is also comparable to two-stage approaches where batch correction is performed prior to dimension reduction. These approaches include Combatting atch Effects (ComBat, Johnson et al., 2007), fastMNN (Haghverdi et al., 2018), and Harmony (Korsunsky et al., 2019) which are all widely employed and have been compared recently (Tran et al., 2020; Luecken et al., 2022; Ryu et al., 2023). For our analyses, we compare p-*t*-SNE and p-UMAP to performing ComBat, fastMNN, or Harmony followed by *t*-SNE or UMAP. ComBat is run using the R implementation in the ComBatHarmonization package (https://github.com/Jfortin1/ComBatHarmonization). fastMNN is run with default settings using the batchelor package version 1.19.1 in R. Harmony is performed with default settings on the top 20 principal components using the harmony package version 1.2.0.”

The results of this analysis are summarized in **Section 4.1**, citing new **Supplementary Figures 1 and 2**:

“Compared to batch correction using ComBat, fastMNN, or Harmony followed by dimension reduction, p-*t*-SNE and p-UMAP w.r.t. study demonstrate comparable performance but fastMNN+*t*-SNE and fastMNN+UMAP outperform other methods (**Supplementary Figures 1 and 2**)”

In the **Discussion**, we comment on these findings:

“As an alternative for batch correction, harmonization methods have been extensively used to reduce batch-related confounding in visualization and downstream analyses (Tran et al., 2020; Luecken et al., 2022; Ryu et al., 2023). Compared to harmonization followed by dimension reduction, we find that PAREs can underperform fastMNN followed by *t*-SNE or UMAP. However, PAREs have several use cases beyond batch adjustment since PAREs can handle continuous confounding variables and multiple confounders.”

Also in the abstract: “Our results show that the PARE framework can remove batch effects in single-cell sequencing.” Evidence is only provided for visualisation, not other downstream applications. Best to add “for reduced dimensional visualisation” or similar, to be specific.

Thank you for this suggestion. We agree, and have changed that sentence to read “For lower-dimensional visualization, our results show that the PARE framework can remove batch effects in single-cell sequencing data as well as separate clinical and technical variability in neuroimaging measures.”

The metric “LISI” used will need to be explained in more depth and justified within the manuscript. Are there other metrics, what are the pros and cons, why was this metric in particular chosen? All comparisons in the work rely on this metric. This information is missing from the manuscript and the reader is merely referred to a citation, which is not sufficient.

We agree that the current justification and choice of metric are insufficient for this analysis. As a result, we now compute the average silhouette width (ASW) for our primary analyses, which is a distance-based metric that complements the neighborhood-based LISI statistic. For clarification and justification, we highlight that the LISI and ASW have been effectively used in recent papers to compare batch correction methods. In **Section 3.1**, we describe our additional analysis:

“We compare our methods visually and numerically using the local inverse Simpson’s index (LISI, Korsunsky et al., 2019) and the average sillouette width (ASW, Rousseeuw, 1987). Recent work has demonstrated that these metrics can effectively evaluate performance of modern batch correction methods (Tran et al., 2020; Luecken et al., 2022). For measuring integration of cells across batches, we compute LISI for batch (bLISI), which captures the effective number of batches in a local neighborhood around each cell. We also examine LISI computed for cell type (cLISI), which captures the number of neighboring cell types and decreases as the separation between cell types increases. We compute bLISI and cLISI across a range of perplexity values, which capture different neighborhood sizes. Additionally, we compute the ASW for batch (bASW), which is a distance-based statistic that captures the average distance of one cell to cells of its own batch versus other batches. Lower bASW indicates better batch correction performance. For cell type, we similarly compute ASW for cell type (cASW) where higher cASW indicates better separation between cell types.”

In **Section 4.1**, we report the new findings using ASW, “Applying p-*t*-SNE and p-UMAP to remove batch effects considerably reduces separation by batch both visually and numerically (**Figure 1**), as measured by increases in bLISI and decreases in bASW for p-UMAP (UMAP median bASW = 0.22 for UMAP, p-UMAP w.r.t. study median bASW = =0.06)”

Finally in the **Discussion**, we compare results from these two statistics and note as a limitation that future studies could consider other statistics as well:

“Throughout our case studies, we perform computational evaluations using the neighborhood-based LISI and distance-based ASW statistics, which are two separate approaches for evaluating separation between categories. In our scRNA-seq analyses, the LISI and ASW generally provide congruent results. However, we note that there are other statistics which will be considered in future studies, including several metrics that have been compared to the LISI and ASW across a larger number of scRNA-seq datasets (Tran et al., 2020; Luecken et al., 2022)”

The figure captions are merely descriptive of the plots and lack insight and/or conclusions (take home message for the reader). These need to be improved.

Thank you for this suggestion. We’ve considerably revised all of the figure captions to include take-home messages and clearer descriptions.

There does not seem to be a link to the code on GitHub (or similar). I have not managed to find the code by Googling the name of the authors or title of the manuscript. Please make your software available.

Thank you for pointing this out. We have now included code for implementation of PARE along with a vignette that contains example calls at <https://github.com/andy1764/PARE>. This is linked in the “Availability of data and materials” section. The software is flexible to the choice of dimension reduction method, which is demonstrated in our vignette: <https://github.com/andy1764/PARE/blob/main/vignettes/pare.Rmd>

**References**

Chari, T., & Pachter, L. (2023). The specious art of single-cell genomics. *PLOS Computational Biology*, *19*(8), e1011288.

Wang, Y., Sun, F., Lin, W., & Zhang, S. (2022). AC-PCoA: Adjustment for confounding factors using principal coordinate analysis. *PLOS Computational Biology*, *18*(7), e1010184.
